# Clinical Evidence and Proposed Mechanisms for Cardiovascular and Kidney Benefits from Sodium–Glucose Co-transporter-2 Inhibitors

**DOI:** 10.17925/EE.2022.18.2.106

**Published:** 2022-11-29

**Authors:** Joshua J Neumiller, Fredrick J Lienhard, Radica Z Alicic, Katherine R Tuttle

**Affiliations:** 1. College of Pharmacy and Pharmaceutical Sciences, Washington State University, Spokane, WA, USA; 2. Providence Medical Research Center, Providence Health and Services, Spokane, WA, USA; 3. University of Washington School of Medicine, University of Washington, Spokane and Seattle, WA, USA; 4. Nephrology Division, Kidney Research Institute, and Institute of Translational Health Sciences, University of Washington, Spokane and Seattle, WA, USA

**Keywords:** Atherosclerotic cardiovascular disease, sodium glucose cotransporter-2 inhibitors, cardiovascular outcome trials, diabetic kidney disease, heart failure, type 2 diabetes

## Abstract

The number of people living with type 2 diabetes (T2D) and its complications worldwide is increasing at an alarming rate. Fortunately, our understanding of the benefits of glucose-lowering agents from the sodium–glucose co-transporter-2 (SGLT2) inhibitor and glucagon-like peptide-1 (GLP-1) receptor agonist classes on cardiovascular and kidney outcomes is advancing; this means we now have new options to mitigate the risks of these complications in patients with T2D. The SGLT2 inhibitors have consistently demonstrated benefits on atherosclerotic cardiovascular disease (ASCVD), chronic kidney disease (CKD) and heart failure (HF) events in dedicated outcome trials. Large guidelines groups now recommend SGLT2 inhibitors as a standard of care in patients with T2D and comorbid ASCVD, CKD and/ or HF. Evolving evidence additionally indicates kidney and HF benefits of SGLT2 inhibitors in populations without diabetes. These agents likely provide heart and kidney benefits through multiple mechanisms, as their impact on heart and kidney outcomes cannot be fully explained by their direct metabolic effects. On-going work to elucidate the beneficial mechanisms at play with SGLT2 inhibitors will help further optimize these life-saving therapies in patients with and without T2D.

Over 37 million people in the USA live with diabetes mellitus, equating to over 11% of the population.^1^ The large majority of these individuals (around 90–95%) have type 2 diabetes (T2D).^1^ Approximately 40% of patients T2D develop chronic kidney disease (CKD), with CKD in diabetes being the leading cause of kidney failure and need for kidney-replacement therapy in the USA.^2^ CKD in diabetes is additionally associated with increased risk of heart failure (HF), infections and all-cause and cardiovascular-related death.^2–6^ The presence of either HF or CKD in diabetes is independently associated with increased cardiovascular and all-cause mortality, with risks further amplified by their combined presence: a condition often referred to as cardiorenal disease.^7^ For people living with T2D, optimizing glycaemic control and managing modifiable risk factors (e.g. blood pressure, obesity, cholesterol and smoking cessation) were considered standard-of-care interventions to prevent and/or delay the progression of diabetes-related complications.^8^ Unfortunately, data from the US Centers for Disease Control and Prevention indicate that only about 18% of adults meet combined glycaemic (glycated haemoglobin A1c [HbA1c] <7.0%), blood pressure (<140/90 mmHg), cholesterol (non-high density lipoprotein cholesterol <130 mg/dL) and smoking cessation goals.^1^ Opportunities clearly exist to improve the holistic care for many of our patients with T2D. Fortunately, recently completed cardiovascular and kidney outcomes trials have identified agents from the sodium–glucose co-transporter-2 (SGLT2) inhibitor and glucagon-like peptide-1 receptor agonist (GLP-1 RA) classes as having important benefits on cardiovascular, kidney and HF outcomes in patients with T2D. Accordingly, agents from these medication classes are recommended to improve glycaemic control, promote weight loss and mitigate heart and kidney risk in patients with T2D.^8^

This review discusses current evidence for use of SGLT2 inhibitors based upon available data from large cardiovascular outcome trials, and then discusses dedicated kidney and HF trials. We then briefly discuss the plausible biological mechanisms by which SGLT2 inhibitors convey heart and kidney protection. Additionally, we provide an overview of current clinical practice recommendations for the use of SGLT2 inhibitors to improve glycaemic, heart and kidney outcomes, and discuss important risk-mitigation strategies when initiating these therapies.

**Table 1:** Summary of cardiovascular outcome trials with sodium–glucose co-transporter-2 inhibitors^12–15^

Trial	CANVAS Program (n=10,142)	DECLARE-TIMI 58 (n=17,160)	EMPA-REG OUTCOME (n=7,020)	VERTIS CV (n=8,246)
Treatment	Canagliflozin versus placebo	Dapagliflozin versus placebo	Empagliflozin versus placebo	Ertugliflozin versus placebo
Mean participant age (years)	63	64	63	64
Key inclusion criteria	T2D Pre-existing CVD at ≥30 years of age OR >2 cardiovascular risk factors at ≥50 years of age	T2D Established ASCVD or multiple cardiovascular risk factors	T2D Pre-existing CVD	T2D Established ASCVD
Prior CVD (%)	66	41	>99	100
Mean baseline HbA1c (%)	8.2	8.3	8.1	8.2
Baseline metformin use (%)	77	82	74	76
Median follow-up (years)	3.6	4.2	3.1	3.5
Primary outcome(s)				
HR (95% CI)	Three-point MACE 0.86 (0.75–0.97)	Three-point MACE 0.93 (0.84–1.03)	Three-point MACE 0.86 (0.74–0.99)	Three-point MACE 0.97 (0.85–1.11)
		CV death or HF hospitalization 0.83 (0.73–0.95)		
Key secondary outcomes				
HF hospitalization; HR (95% CI)	0.67 (0.52–0.87)	0.73 (0.61–0.88)	0.65 (0.50–0.85)	0.70 (0.54–0.90)
Worsening nephropathy; HR (95% CI)*	0.60 (0.47–0.77)	0.53 (0.43–0.66)	0.61 (0.53–0.70)	0.81 (0.63–1.04)
Cardiovascular death; HR (95% CI)	0.87 (0.72–1.06)	0.98 (0.82–1.17)	0.62 (0.49–0.77)	0.92 (0.77–1.11)
All-cause mortality; HR (95% CI)	0.87 (0.74–1.01)	0.93 (0.82–1.04)	0.68 (0.57–0.82)	0.93 (0.80–1.08)

*Definitions of worsening nephropathy differed between trials.

*ASCVD = atherosclerotic cardiovascular disease; CI = confidence interval; CVD = cardiovascular disease; HbA1c = glycated haemoglobin A1c; HF = heart failure; HR = hazard ratio; MACE = major adverse cardiovascular event; T2D = type 2 diabetes mellitus.*

## Cardiovascular outcome trials with sodium–glucose co-transporter-2 inhibitors

In 2008, the US Food and Drug Administration (FDA) issued guidance to industry for the evaluation of cardiovascular risk with new glucose-lowering therapies.^9^ The guidance was issued in the wake of cardiovascular safety concerns raised for the thiazolidinedione agent rosiglitazone,^10^ and called for cardiovascular safety and risk to be assessed thoroughly during drug development.^9^ Subsequently, large cardiovascular outcome trials have been conducted and published with medications from the SGLT2 inhibitor, GLP-1 RA and dipeptidyl peptidase-4 (DPP-4) classes. While designed primarily to demonstrate cardiovascular safety, many of the trials conducted with SGLT2 inhibitors and GLP-1 RAs have fortuitously demonstrated cardiovascular benefit relative to placebo, thus ushering in a new paradigm for the use of these agents to not only lower glucose, but to also modify cardiovascular risk.^11^ Results from large cardiovascular outcome trials have been reported for all four SGLT2 inhibitors currently marketed in the USA: canagliflozin, dapagliflozin, empagliflozin and ertugliflozin.^12–15^ Not only have these trials provided foundational knowledge substantiating the cardiovascular benefit of these agents in patients with T2D,^12–15^ importantly, they also included prespecified secondary outcomes that have prompted additional inquiry into the benefits of this class on both kidney and HF outcomes. These cardiovascular outcome trials for each agent are briefly summarized below, and in *Table 1*.

### Canagliflozin

The Canagliflozin Cardiovascular Assessment Study (CANVAS) Program was one of the first cardiovascular outcome trial programmes initiated following the 2008 FDA guidance.^9^ The programme comprised two integrated analyses – CANVAS (initiated in 2009) and CANVAS-Renal (initiated in 2014) – to assess the impact of canagliflozin on cardiovascular, kidney and safety outcomes in participants with T2D.^12^ The primary outcome for the CANVAS programme was a three-point major adverse cardiovascular event (MACE) outcome including of death from cardiovascular causes, non-fatal myocardial infarction (MI) or non-fatal stroke. Key secondary outcomes included progression of albuminuria, and a composite kidney outcome including sustained 40% reduction in estimated glomerular filtration rate (eGFR), kidney-replacement therapy and renal death.^12^

Over 10,000 participants with T2D and high cardiovascular risk were included in the trial and randomized to receive either canagliflozin or placebo.^12^ Canagliflozin treatment was associated with a lower rate of MACE events compared with placebo (26.9 versus 31.5 participants per 1,000 patient-years, respectively), equating to a 14% risk reduction for the primary outcome (hazard ratio [HR] 0.86; 95% confidence interval [CI] 0.75–0.97; p=0.02 for superiority).^12^ This finding supported the FDA’s granting an expanded indication for canagliflozin to reduce the risk for MACE in adults with T2D and established cardiovascular disease.^16^

Benefits on key secondary outcomes were also observed.^12^ For canagliflozin-treated patients, risk was reduced for progression of albuminuria (HR 0.73; 95% CI 0.67–0.79), as well as for the composite kidney outcome (HR 0.60; 95% CI 0.47–0.77). In addition, the risk for HF hospitalization was reduced by 33% compared with placebo (HR 0.67; 95% CI 0.52–0.87).^12^ Benefits observed on secondary kidney and HF outcomes were hypothesis generating, and supported dedicated investigation of canagliflozin in the CKD and HF settings.

In terms of safety, participants receiving canagliflozin experienced adverse reactions generally associated with the class, including volume depletion-related events and genital mycotic infections.^12^ An additional unexpected finding was a two-fold increased event rate for amputations, primarily at the toe or metatarsal.^12^ This finding has raised questions on the general risk of amputation among patients using SGLT2 inhibitors, although any association is currently considered controversial and requires additional surveillance and study. While once included as a black box warning, a recommendation to consider factors that may increase the risk for amputation, including active infections or ulcers on the lower limbs, is now included as a warning in the US Prescribing Information for canagliflozin.^16^

### Dapagliflozin

The Dapagliflozin Effect on Cardiovascular Events—Thrombosis in Myocardial Infarction 58 (DECLARE-TIMI 58) trial was a large cardiovascular outcome trial that enrolled over 17,000 participants with T2D and existing atherosclerotic cardiovascular disease (ASCVD) or its risk factors.^13^ When compared with cardiovascular outcome trials for other SGLT2 inhibitors, DECLARE-TIMI 58 included a smaller fraction of participants with established ASCVD at baseline, representing a lower-risk population overall (*Table 1*). In this trial, the participants were randomized to receive either dapagliflozin or placebo. DECLARE-TIMI 58 included two primary outcomes: three-point MACE, and a cardiovascular composite outcome including cardiovascular death and HF hospitalization. Key prespecified secondary outcomes included all-cause mortality and a kidney composite outcome (≥40% decrease in eGFR to <60 mL/min/1.73 m^2^, progression to end-stage kidney disease, or death from cardiovascular or renal causes).^13^

After a median follow-up of 4.2 years, dapagliflozin was found to be non-inferior to placebo for three-point MACE (HR 0.93; 95% CI 0.84–1.03; p=0.17).^13^ Dapagliflozin treatment did, however, reduce the risk for the co-primary outcome of cardiovascular death or HF hospitalization by 17% compared with placebo (HR 0.83; 95% CI 0.73–0.95; p=0.005). This finding was primarily driven by benefit on HF hospitalization (HR 0.73; 95% CI 0.61–0.88). Supporting the hypothesis of kidney benefit with dapagliflozin treatment, a benefit was observed relative to placebo for the kidney composite secondary outcome (HR 0.76; 95% CI 0.67–0.87). Key safety findings reported in the DECLARE-TIMI 58 trial included increased event rates with dapagliflozin compared with placebo for diabetic ketoacidosis (0.3% versus 0.1%; p=0.02) and genital mycotic infections leading to treatment discontinuation and/or considered to be a serious adverse event (0.9% versus 0.1%; p<0.001). An increased risk for lower-limb amputations was not reported, although it was not included as an event of special interest in this trial.^13^

### Empagliflozin

The Empagliflozin Cardiovascular Outcome Event Trial in Type 2 Diabetes Mellitus Patients (EMPA-REG OUTCOME) assessed the impact of empagliflozin versus placebo on cardiovascular morbidity and mortality in participants with T2D and established cardiovascular disease.^14^ The primary outcome of the trial was a composite of death from cardiovascular disease, non-fatal MI and non-fatal stroke. The primary outcome occurred in 10.5% of participants in the empagliflozin group and 12.1% of participants in the placebo group (HR 0.86; 95% CI 0.74–0.99; p=0.04 for superiority).^14^ When examining components of the primary outcome, a 38% risk reduction for cardiovascular death and a 32% risk reduction for all-cause mortality was observed with empagliflozin.^14^ These findings supported the FDA’s granting empagliflozin an indication to reduce the risk of cardiovascular death in patients with T2D and established cardiovascular disease.^18^

Analyses of EMPA-REG OUTCOME participants with CKD at baseline (eGFR <60 mL/min/1.73 m^2^ and/or urine albumin-to-creatinine ratio [UACR] >300 mg/g) found that empagliflozin treatment improved clinical outcomes and reduced mortality in this population.^19^ Participants receiving empagliflozin had a higher event rate for genital mycotic infections, but no increases in other adverse events relative to placebo were observed.^14^

### Ertugliflozin

The Evaluation of Ertugliflozin Efficacy and Safety Cardiovascular Outcomes Trial (VERTIS CV) is the only trial to date to have published outcomes with ertugliflozin.^15^ VERTIS CV enrolled over 8,000 participants with T2D and established ASCVD. Participants received treatment with either ertugliflozin or placebo and were followed for a mean 3.5 years. The primary three-point MACE outcome occurred in 11.9% of participants in both the treatment and placebo groups (HR 0.97; 95% CI 0.85–1.11; p<0.001 for non-inferiority).^15^

While ertugliflozin was not found to be superior to placebo for the primary outcome, several secondary and exploratory outcomes suggested a benefit of ertugliflozin treatment. Consistent with cardiovascular outcome trials for other SGLT2 inhibitors, ertugliflozin was associated with a reduction in risk for HF hospitalization versus placebo (HR 0.70; 95% CI 0.54–0.90).^15^ Additionally, an analysis of exploratory kidney outcomes suggested ertugliflozin treatment reduced the risk for a composite kidney outcome (including a sustained 40% decline in eGFR from baseline, progression to kidney-replacement therapy or renal death), and also preserved eGFR and reduced UACR.^20^ The most common adverse events noted during the trial were consistent with cardiovascular outcome trials for other SGLT2 inhibitors and included genital mycotic and urinary tract infections.^15^

## Dedicated kidney outcome trials with sodium–glucose co-transporter-2 inhibitors

Following the hypothesis-generating findings from cardiovascular outcome trials suggesting kidney benefits with SGLT2 inhibitors, several dedicated kidney outcome trials have been conducted to assess these potential benefits in detail. To date, full results are available from three dedicated kidney outcome trials with canagliflozin, dapagliflozin and sotagliflozin (*Table 2*).^21–23^

The first of these to be published was the Canagliflozin and Renal Events in Diabetes with Established Nephropathy Clinical Evaluation (CREDENCE) trial.^21^ CREDENCE enrolled patients with T2D and albuminuria (UACR >300 mg/g). The trial was stopped early following a planned interim analysis due to overwhelming efficacy. Risk for the primary kidneyspecific composite outcome (end-stage kidney disease, doubling of serum creatinine, or cardiovascular or renal death) was reduced by 30% with canagliflozin treatment versus placebo. Canagliflozin also improved several cardiovascular composite outcomes compared with placebo, including composites of cardiovascular death or HF hospitalization (HR 0.69, 95% CI 0.57–0.83; p<0.01), and of cardiovascular death, MI or stroke (HR 0.80; 95% CI 0.67–0.95; p=0.01). The risk of HF hospitalization was also reduced with SGLT2 inhibition when compared with placebo (HR 0.61; 95% CI 0.47–0.80; p<0.001).^21^

**Table 2:** Summary of kidney outcome trials with sodium–glucose co-transporter-2 inhibitors^21–23^

Trial	CREDENCE (n=4,401)	DAPA-CKD (n=4,304)	SCORED (n=10,584)
Treatment	Canagliflozin versus placebo	Dapagliflozin versus placebo	Sotagliflozin versus placebo
Mean participant age (years)	63	62	69
Key inclusion criteria	T2D eGFR 30 to <90 mL/min/1.73 m^2^ UACR >300 to 5,000 mg/g Treated with RAS inhibitor for ≥4 weeks prior to randomization	eGFR 25–75 mL/min/1.73 m^2^ UACR of 200–5,000 mg/g Treated with RAS inhibitor for ≥4 weeks prior to screening	T2D eGFR 25–60 mL/min/1.73 m^2^ Presence of ≥1 additional cardiovascular risk factor
Baseline diagnosis of T2D (%)	100	67	100
Mean baseline HbA1c (%)	8.3	7.1	8.3
Baseline metformin use (%)	58	29	55
Median follow-up (years)	2.6	2.4	1.3
Primary outcome			
HR (95% CI)	End-stage kidney disease, doubling of serum creatinine, or renal or cardiovascular death 0.70 (0.59–0.82)	≥50% decline in eGFR, end-stage kidney disease, or renal or cardiovascular death 0.61 (0.51–0.72)	Total number of cardiovascular deaths, HF hospitalizations, or urgent visits for HF 0.74 (0.63–0.88)
Key secondary outcomes			
Progression to end-stage kidney disease; HR (95% CI)	0.68 (0.54–0.86)	0.64 (0.50–0.82)	N/R
Cardiovascular death; HR (95% CI)	0.78 (0.61–1.00)	0.81 (0.58–1.12)	0.90 (0.73–1.12)
All-cause mortality; HR (95% CI)	0.83 (0.68–1.02)	0.69 (0.53–0.88)	0.99 (0.83–1.18)

*CI = confidence interval; CVD = cardiovascular disease; eGFR = estimated glomerular filtration rate; HbA1c = glycated haemoglobin A1c; HR = hazard ratio; N/R = not reported; RAS = renin-angiotensin system; T2D = type 2 diabetes mellitus; UACR = urinary albumin-to-creatinine ratio.*

The Dapagliflozin and Prevention of Adverse Outcomes in Chronic Kidney Disease (DAPA-CKD) trial similarly assessed the efficacy and safety of dapagliflozin versus placebo in patients with CKD.^22^ Expanding on the findings from CREDENCE, DAPA-CKD enrolled patients with CKD and albuminuria (UACR ≥200 mg/g) with or without T2D (*Table 2*). As with CREDENCE, DAPA-CKD was also halted early following an Independent Data Monitoring Committee recommendation based on an overwhelming signal of benefit. The primary composite kidney outcome – a sustained decline in eGFR of at least 50%, progression to end-stage kidney disease or death from cardiovascular or renal causes – was significantly reduced with dapagliflozin treatment compared with placebo (HR 0.61; 95% CI 0.51–0.72; p<0.001).^22^

Results from the Effect of Sotagliflozin on Cardiovascular and Renal Events in Patients with Type 2 Diabetes and Moderate Renal Impairment Who Are at Cardiovascular Risk (SCORED) trial were reported in 2021.^23^ While sotagliflozin is not approved for use in the USA, the SCORED trial contributes to the growing body of evidence that SGLT2 inhibitors are beneficial for cardiovascular and kidney outcomes in patients with T2D and CKD. The trial was ended early due to loss of funding, and thus its primary outcome was changed to compare the total number of cardiovascular deaths, HF hospitalizations and urgent visits for HF between sotagliflozin and placebo (*Table 2*).^23^ With regard to kidney outcomes, a kidney composite secondary outcome (including ≥50% decline in eGFR from baseline for ≥30 days, long-term dialysis, kidney transplant or sustained eGFR <15 mL/min/1.73 m^2^ for ≥30 days) trended toward benefit for SGLT2 inhibition (HR 0.71; 95% CI 0.46–1.08).^23^

While its full results have not been published at the time of this writing, high-level reported findings from The Study of Heart and Kidney Protection With Empagliflozin (EMPA-KIDNEY) have been positive.^24^ As with CREDENCE and DAPA-CKD, the trial was stopped early due to clear benefit during a planned interim analysis. EMPA-KIDNEY enrolled over 6,500 participants with CKD with or without T2D.^25,26^ Participants were included if they had an eGFR of ≥20 to <45 mL/min/1.73 m^2^ with or without albuminuria, or eGFR ≥45 to <90 mL/min/1.73 m^2^ with albuminuria (UACR ≥200 mg/g). Based on its inclusion criteria, EMPA-KIDNEY will provide important data on the impact of empagliflozin on kidney outcomes in patients with low eGFR and wide-ranging albuminuria. The primary outcome is a kidney composite including time to first occurrence of end-stage kidney disease, a sustained decline in eGFR to <10 mL/min/1.73 m^2^, sustained decline of ≥40% in eGFR from baseline, or cardiovascular or renal death.^25,26^

Importantly, CREDENCE, DAPA-CKD and EMPA-KIDNEY all evaluated the effect of SGLT2 inhibitor treatment on top of standard-of-care angiotensin-converting enzyme (ACE) inhibitor or angiotensin II receptor blocker (ARB) therapy.^21,22,26^ The benefits observed within these kidney outcome trials therefore demonstrate that SGLT2 inhibitors address residual risk for progression of kidney disease despite optimized ACE inhibitor or ARB treatment. For this reason, SGLT2 inhibitors are now considered standard of care for patients with T2D and CKD, as discussed in more detail later.

## Dedicated heart failure outcome trials with sodium–glucose co-transporter-2 inhibitors

As discussed above, cardiovascular and kidney outcome trials with SGLT2 inhibitors have consistently reported benefits on HF outcomes (*Table 1* and *Table 2*). These findings have been substantiated by several large HF outcome trials published to date with dapagliflozin, sotagliflozin and empagliflozin (*Table 3*).^27–31^

**Table 3:** Summary of heart failure outcome trials with sodium–glucose co-transporter-2 inhibitors^27–31^

Trial	DAPA-HF (n=4,744)	DELIVER (n=6,263)	EMPEROR-Reduced (n=3,730)	EMPEROR-Preserved (n=5,988)	SOLOIST-WHF (n=1,222)
Treatment	Dapagliflozin versus placebo	Dapagliflozin versus placebo	Empagliflozin versus placebo	Empagliflozin versus placebo	Sotagliflozin versus placebo
Mean participant age (years)	66	72	67	72	70
Key inclusion criteria	NYHA class II, III or IV HF Ejection fraction ≤40%	Stabilized HF Ejection fraction >40%	NYHA class II, III or IV HF Ejection fraction ≤40%	NYHA class II, III or IV HF Ejection fraction >40%	T2D HF hospitalization and treatment with intravenous diuretic therapy
Baseline diagnosis of T2D (%)	42	45	50	49	100
Prior HF (%)	100	100	100	100	100
Median follow-up (years)	1.5	2.3	1.3	2.2	0.75
Primary outcome					
HR (95% CI)	Worsening HF or cardiovascular death 0.74 (0.65–0.85)	Worsening HF, cardiovascular death or urgent visit for HF 0.82 (0.73–0.92)	Cardiovascular death or HF hospitalization 0.75 (0.65–0.86)	Cardiovascular death or HF hospitalization 0.79 (0.69–0.90)	Cardiovascular death or hospitalization or urgent visit for HF 0.67 (0.52–0.85)
Key secondary outcomes					
HF hospitalization; HR (95% CI)	0.70 (0.59–0.83)	0.77 (0.67–0.89)	0.69 (0.59–0.81)	0.71 (0.60–0.83)	0.64† (0.49–0.83)
Worsening nephropathy; HR (95% CI)*	0.71 (0.44–1.16)	N/R	0.50 (0.32–0.77)	0.95 (0.73–1.24)	N/R
Cardiovascular death; HR (95% CI)	0.82 (0.69–0.98)	0.88 (0.74–1.05)	0.92 (0.75–1.12)	0.91 (0.76–1.09)	0.84 (0.58–1.22)
All-cause mortality; HR (95% CI)	0.83 (0.71–0.97)	0.94 (0.83–1.07)	0.92 (0.77–1.10)	1.00 (0.87–1.15)	0.82 (0.59–1.14)

*Definitions of worsening nephropathy differed between trials.

*†Included both HF hospitalization and urgent visits for HF.*

*CI = confidence interval; HF = heart failure; HR = hazard ratio; N/R = not reported; NYHA = New York Heart Association; T2D = type 2 diabetes mellitus.*

The Dapagliflozin and Prevention of Adverse Outcomes in Heart Failure (DAPA-HF) trial enrolled over 4,700 participants with New York Heart Association (NYHA) class II–IV HF and an ejection fraction ≤40%.^27^ This HF with reduced ejection fraction population included participants with and without T2D. The primary outcome was a composite of worsening HF (hospitalization or an urgent care visit resulting in intravenous therapy for HF) or cardiovascular death. The primary outcome occurred in 16.3% and 21.2% of participants in the dapagliflozin and placebo groups, respectively (HR 0.74; 95% CI 0.65–0.85; p<0.001). Treatment with dapagliflozin was similarly beneficial in those with and without T2D.^27^

As did DAPA-HF, the Empagliflozin Outcome Trial in Patients with Chronic Heart Failure and a Reduced Ejection Fraction (EMPEROR-Reduced) trial enrolled participants (n=3,730) with NYHA class II–IV HF and an ejection fraction ≤40%.^29^ Approximately half of participants in each treatment group (empagliflozin or placebo) had a baseline diagnosis of T2D. The primary outcome was a composite of cardiovascular death or hospitalization for worsening HF. The primary outcome occurred in 19.4% of participants in the empagliflozin group and in 24.7% of participants in the placebo group (HR 0.75; 95% CI 0.65–0.86; p<0.001).^29^ Again, benefit for the primary outcome was consistent regardless of T2D status. The investigators also reported a decline in eGFR over the course of the trial, but with a slower annual decline among those treated with empagliflozin than those treated with placebo (-0.55 versus -2.28 mL/min/1.73 m^2^/year, respectively; p<0.001).^29^

The Empagliflozin Outcome Trial in Patients with Chronic Heart Failure with Preserved Ejection Fraction (EMPEROR-Preserved) trial expanded the investigation of SGLT2 inhibitors into a population with NYHA class II–IV HF with preserved ejection fraction (>40%).^30^ Nearly 6,000 participants were enrolled in the trial, and less than half of them had T2D at baseline. The primary outcome was a composite of cardiovascular death or hospitalization for HF, and empagliflozin treatment significantly lowered the risk for the primary outcome compared with placebo (HR 0.79; 95% CI 0.69–0.90; p<0.001). Adverse events reported more frequently in this population with SGLT2 inhibition than with placebo included genital mycotic infections, urinary tract infections and hypotension.^30^

Results from the Dapagliflozin Evaluation to Improve the Lives of Patients with Preserved Ejection Fraction Heart Failure (DELIVER) trial provide additional evidence of benefit in patients with preserved ejection fraction.^28^ DELIVER reported a benefit of dapagliflozin therapy versus placebo on the primary composite outcome of worsening HF (unplanned hospitalization or an urgent visit for HF) or cardiovascular death (HR 0.82; 95% CI 0.73–0.92; p<0.001; *Table 3*).^28^

The Effect of Sotagliflozin on Cardiovascular Events in Patients with Type 2 Diabetes Post Worsening Heart Failure (SOLOIST-WHF) adds to the studies described above by assessing the impact of SGLT2 inhibition on HF outcomes in patients shortly following an episode of decompensated HF.^31^ Participants enrolled in the trial had a diagnosis of T2D and were hospitalized and treated with intravenous diuretics for HF symptoms. The first dose of sotagliflozin or placebo was administered prior to hospital discharge in nearly 49% of participants, and to the rest a median of 2 days following discharge.^31^ The primary outcome was a composite that included cardiovascular death, and hospitalizations and urgent visits for HF. Sotagliflozin treatment decreased risk for the primary endpoint by 33% compared with placebo (HR 0.67; 95% CI 0.52–0.85; p<0.001).^31^

A recently published meta-analysis of the large HF outcome trials summarized in *Table 3* confirms that SGLT2 inhibitor therapy reduces the risk of cardiovascular death and HF hospitalization in a broad range of patients with HF.^32^ Based on their findings, the authors concluded that current evidence supports the foundational use of SGLT2 inhibitors to manage HF, irrespective of ejection fraction or care setting.^32^

## Proposed mechanisms for cardiovascular and kidney benefit with sodium–glucose co-transporter-2 inhibition

T2D is associated with a chronic state of inflammation and measurable increases in markers of inflammation (e.g. interleukins, sialic acid and C-reactive protein) and circulating immune cells.^33–39^ It is a state of nutrient excess in which activation of nutrient-deprivation sensors is decreased, including sirtuin-1 (SIRT1), adenosine monophosphate-activated protein kinase (AMPK) and hypoxia-inducible factors (HIFs; especially HIF-2α).^40–42^ Suppressed activation of SIRT1 and AMPK contributes to the development of glomerular hyperfiltration, promotes mitochondrial dysfunction, oxidative stress and excess inflammation, and reduces autophagic clearance of damaged organelles.^40^ This suppressed SIRT1/AMPK signalling and resulting pro-inflammatory state, has deleterious effects on a variety of organ systems and contributes to the development of kidney and heart complications in diabetes.^41–43^

The pathogenesis of cardiovascular disease in T2D is driven by a multitude of factors. Metabolic, haemodynamic and inflammatory derangements result in cellular and functional changes in the myocardium. For example, dysfunction of the endothelium, progression of atherosclerosis, hypertrophy of cardiac myocytes and myocardial fibrosis are all key changes that are partly driven by inflammation in patients with T2D.^44^ These structural changes subsequently lead to changes in ventricular function, altering the perfusion of tissues supplied by both small and large vessels.^44^ The altered metabolic state also contributes to impaired substrate switching and its resulting energetic deficits in the heart.^45^

The pathogenesis of CKD in T2D is likewise associated with metabolic and haemodynamic changes resulting from chronic inflammation, progressive kidney fibrosis and progressive eGFR decline. As previously noted, T2D is associated with an increased state of inflammation; oxidative stress and increased production of advanced glycation endproducts serve to activate the immune system early in the natural history of T2D.^34,46,47^ Indeed, increases in inflammatory markers have been observed in the urine of patients with early T2D and nephropathy.^48,49^ Within kidney tissue, changes include infiltration of inflammatory cells and activation of resident T-cell populations.^50^ Up-regulation of pro-fibrotic cytokines further contributes to kidney fibrosis and destruction of the parenchyma.^47,51,52^ Matrix protein production is increased in kidney mesangial cells, compounded by a diminished breakdown of fibrotic proteins by matrix metalloproteinases.^53^

**Figure 1:**
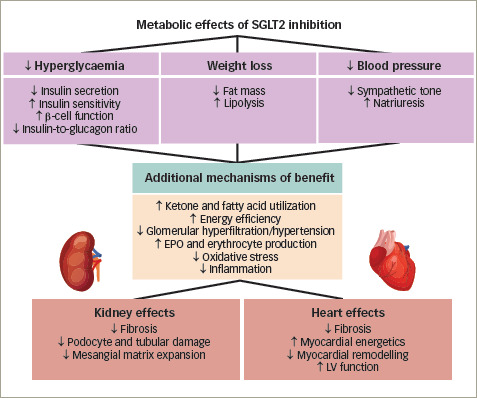
Putative mechanisms of sodium–glucose co-transporter-2 inhibitor therapies on kidney and cardiovascular disease^50–52^

SGLT2 inhibitors are proposed to mitigate heart and kidney risk through multiple mechanisms (*Figure 1*).^54–56^ When used clinically, SGLT2 inhibitors are associated with reductions in glycaemia, weight and blood pressure, and with ketogenesis and erythrocytosis.^54^ The glycaemic effects of SGLT2 inhibition (the indication for which this class of medications was developed) do not fully account for the organ benefits of SGLT2 inhibitors observed in outcome trials, especially when considering the benefits in participants without diabetes that have been observed in several HF and kidney outcome trials.^22,24,27–30^ Further, evidence from large T2D trials suggests that intensifying glycaemic control does not notably reduce patients’ risk for major ASCVD events.^57–59^ Accordingly, the cardiovascular and kidney benefits observed in large outcome trials with SGLT2 inhibitors are not fully explained by their metabolic effects.

*Sodium–glucose co-transporter-2 (SGLT2) inhibitors block reabsorption of glucose in the proximal tubule with resulting increases in urinary glucose disposal and mild osmotic diuresis and natriuresis. SGLT2 inhibitors decrease blood glucose levels and are associated with decreases in insulin secretion, improved* β*-cell function, and improvements in insulin resistance. Blood pressure is improved via mild diuresis/ natriuresis and a reduction in sympathetic tone. Net calorie loss with SGLT2 inhibition is ∼200–300 kcal/day mimicking a fasting-like metabolism. Net calorie loss provokes whole-body adaptive responses in energy metabolism by activation of sirtuin-1 and adenosine monophosphate-activated protein kinase with shifts of energy substrates from carbohydrates to fatty acids and ketone bodies. The achieved fasting-like metabolism improves mitochondrial dynamics and oxidative stress. Mimicking systemic hypoxia, SGLT2 inhibition upregulates hypoxia-inducible factors (HIFs; especially HIF-2*α*) resulting in erythropoietin production with an associated elevation of haematocrit, which may ameliorate organ oxygen delivery. SGLT2 inhibition additionally has anti-inflammatory effects, reduces oxidative stress and apoptosis, and increases autophagy. These changes result in reduced heart and kidney inflammation, fibrosis and structural damage.*

*EPO = erythropoietin; LV = left ventricular; SGLT2 = sodium–glucose co-transporter-2.*

**Figure 2:**
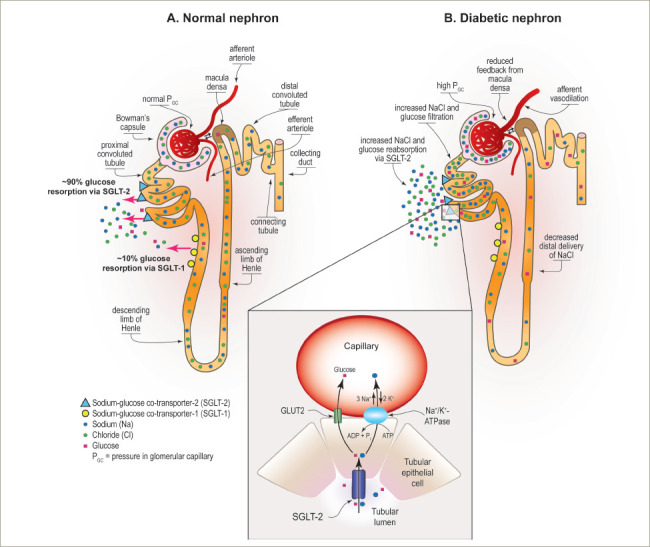
Glucose reabsorption via sodium–glucose co-transporter-1 and -2 in (A) non-diabetic and (B) diabetic kidneys

One likely mechanism of benefit of SGLT2 inhibition in the setting of CKD is by normalizing glomerular haemodynamics by restoring tubulo-glomerular feedback. Glomerular hyperfiltration is an early haemodynamic change observed in at least 40% of patients with T2D (*Figure 2*).^54^ It is driven by multiple factors including hyperglycaemia, hyperaminoacidaemia and increased proximal tubular reabsorption of sodium and glucose via SGLT2 and SGLT1 (*Figure 2*).^54^ Tubulo-glomerular feedback is a mechanism through which reabsorption of sodium and chloride in the macula densa promotes the release of adenosine (*Figure 2*), which then constricts the afferent arterioles in the glomerulus.^54^ Increased reabsorption of sodium and chloride in the proximal tubule diminishes the delivery of solutes to the macula densa, and consequently reduces adenosine production. This causes afferent arteriolar vasodilation, glomerular hyperperfusion and hyperfiltration within the glomerulus.^60^

*Expressed apically in the epithelium of the proximal convoluted tubule, sodium–glucose co-transporter-2 (SGLT2) reabsorbs about 90% of glucose from the urinary filtrate. The remaining 10% is reabsorbed by SGLT1, a high-affinity and low-capacity transporter expressed apically in the epithelium of the straight descending proximal tubule. Reprinted with permission from Alicic RZ, et al. Diabetes. 2019;68:248–57.^54^*

**Figure 3:**
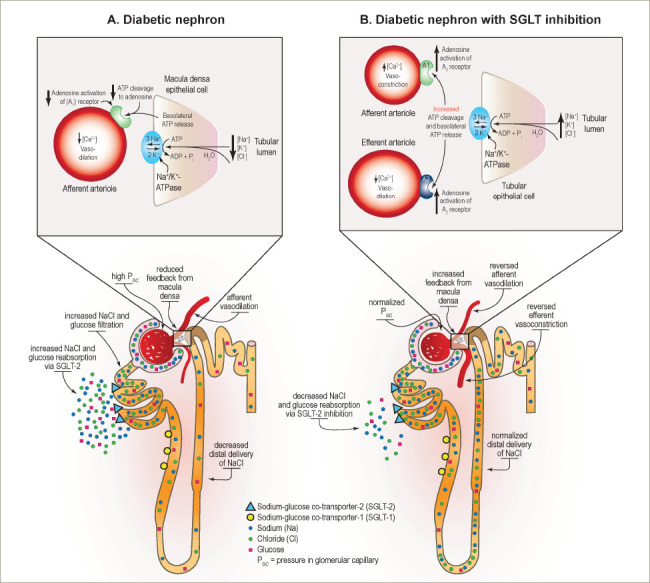
Effects of diabetes and sodium–glucose co-transporter-2 inhibition on nephron haemodynamics

With use of SGLT2 inhibitors, solute delivery to the macula densa is restored by blocking the reabsorption of sodium chloride in the proximal tubule, thus restoring normal tubulo-glomerular feedback (*Figure 3*).^54^ In addition to their effects on tubulo-glomerular feedback, SGLT2 inhibitors also likely convey additional anti-inflammatory, anti-fibrotic, energetic and cardiac remodelling benefits that protect the heart and kidney (*Figure 1*).^54–56^ By inducing a biologic state that mimics starvation, SGLT2 inhibitors reduce nutrient excess signalling and help to reverse the cycle of glomerular hyperfiltration, inflammation and suppressed autophagy-mediated cytoprotection.^42^

## Current recommendations for use of sodium–glucose co-transporter-2 inhibitors to improve cardiovascular and kidney outcomes

SGLT2 inhibitors are highly effective glucose-lowering agents for use in patients with T2D. Multiple large guidelines groups, including the American Diabetes Association (ADA), recommend SGLT2 inhibitors as an option for patients not meeting individualized glycaemic goals to reduce hyperglycaemia and HbA1c.^8,61–63^ SGLT2 inhibitors are also a preferred option for patients with T2D when there is a desire to minimize hypoglycaemia and/or to minimize weight gain or promote weight loss.^8^ Furthermore, the ADA 2022 Standards of Medical Care in Diabetes recommends using SGLT2 inhibitors for their organ protective effects in patients with CKD, ASCVD and/or HF, independent of HbA1c or individualized HbA1c goals.^8^

When considering a glucose-lowering agent in a patient with T2D, the first recommended consideration per the 2022 ADA Standards of Care, and the European Association for the Study of Diabetes (EASD)/European Society of Cardiology (ESC) Consensus Statement is whether the patient has ASCVD, HF or CKD.^8,63^ If a patient has one of these comorbidities, it is recommended to consider incorporating an agent with evidence of benefit into their medication regimen. Within the ADA Standards of Care, agents with ‘proven benefit’ are operationally defined as those with an expanded indication for improving ASCVD, HF or CKD outcomes (*Table 4*).^16,18,64,65^ Based on observed HF benefits in patients with and without T2D, the 2022 heart failure guidelines from the American Heart Association/ American College of Cardiology/Heart Failure Society of America recommend SGLT2 inhibitors within their treatment algorithm to improve HF outcomes.^66^

*A: Increased resorption of glucose by sodium–glucose co-transporter-2 (SGLT2) in the proximal convoluted tubule decreases delivery of solutes to the macula densa. The resulting decrease in adenosine triphosphate release from the basolateral membrane of tubular epithelial cells reduces production of adenosine and induces vasodilation of the afferent arteriole. B: SGLT2 inhibitors restore solute delivery to the macula densa with resulting adenosine activation and reversal of vasodilation of the afferent arteriole. Reprinted with permission from Alicic RZ, et al. Diabetes. 2019;68:248–57.^54^*

For patients with T2D and CKD, use of an SGLT2 inhibitor is preferentially recommended by the ADA, EASD, American Association of Clinical Endocrinologists/American College of Endocrinology, ESC and Kidney Disease: Improving Global Outcomes (KDIGO).^8,61–63,67^ For patients with T2D and CKD with albuminuria (e.g. creatinine ≥200 mg/g), the ADA preferentially recommends an SGLT2 inhibitor with primary evidence of reducing CKD progression.^8^ As previously discussed, both canagliflozin and dapagliflozin have demonstrated benefit in patients with T2D and CKD in dedicated kidney outcome trials, and are indicated for improving CKD outcomes;^21,22^ an indication to improve kidney outcomes is anticipated in the near future based on findings from the EMPAKidney trial.^24^

If a SGLT2 inhibitor cannot be taken due to a contraindication or drug intolerance, the ADA recommends adding a GLP-1 RA with proven cardiovascular benefit.^8^ For patients with T2D and CKD (eGFR <60 mL/min/1.73 m^2^) who do not have albuminuria, the 2022 ADA Standards of Care state that either an SGLT2 inhibitor or GLP-1 RA with proven cardiovascular benefit can be used. These recommendations are based on the considerable cardiovascular-related morbidity and mortality risks that patients with CKD face.^47^

KDIGO similarly recommends a first-line combination glucose-lowering regimen including metformin plus a SGLT2 inhibitor in patients with T2D and CKD, unless contraindicated.^67^ KDIGO recommends an SGLT2 inhibitor for patients with T2D and CKD (eGFR >20 mL/min/1.73 m^2^), irrespective of albuminuria, based on evidence suggesting that SGLT2 inhibitors confer consistent kidney and cardiovascular benefits irrespective of albuminuria.^67^

While the glycaemic, heart and kidney benefits of SGLT2 inhibitors are clear, it remains paramount that these medications are used safely: they are not without risks. SGLT2 inhibitors increase the risk for certain genito-urinary infections and associate with some less common adverse events (such as hypovolaemia–related events and euglycaemic diabetic ketoacidosis) that have been identified in clinical trials and in post-marketing surveillance.^8^ Risk mitigation strategies and patient counselling are thus important to maximize safety with the use of these agents (*Table 5*).^67^ A recent consensus report from the ADA and KDIGO notes that patients with T2D requiring insulin treatment are at particular risk for SGLT2-inhibitor-associated diabetic ketoacidosis; to mitigate the risks they suggest maintaining at least a low dose of insulin and considering holding SGLT2 inhibitor treatment during acute illness.^68^ Ketone screening can additionally be considered (*Table 5*).

**Table 4:** Labelled indications and dosing for currently available sodium–glucose co-transporter-2 inhibitors^16,18,64,65^

	Canagliflozin	Dapagliflozin	Empagliflozin	Ertugliflozin
Availability	100 mg tablets 300 mg tablets	5 mg tablets 10 mg tablets	10 mg tablets 25 mg tablets	5 mg tablets 10 mg tablets
Indication(s)	Adjunct to diet and exercise to improve glycaemic control in adults with T2D To reduce the risk of MACE in adults with T2D and established cardiovascular disease To reduce the risk of end-stage kidney disease, doubling of serum creatinine, cardiovascular death and hospitalization for HF in adults with T2D and diabetic nephropathy with albuminuria	Adjunct to diet and exercise to improve glycaemic control in adults with T2D To reduce the risk of hospitalization for HF in adults with T2D and established cardiovascular disease or multiple cardiovascular risk factors To reduce the risk of cardiovascular death and hospitalization for HF in adults with HF with reduced ejection fraction (NYHA class II–IV) To reduce the risk of sustained eGFR decline, end-stage kidney disease, cardiovascular death and hospitalization for HF in adults with CKD at risk of progression	Adjunct to diet and exercise to improve glycaemic control in adults with T2D To reduce the risk of cardiovascular death in adults with T2D and established cardiovascular disease To reduce the risk of cardiovascular death and hospitalization for HF in adults with HF	Adjunct to diet and exercise to improve glycaemic control in adults with T2D
Kidney dose adjustment per manufacturer in eGFR (mL/min/1.73 m^2^)	eGFR ≥60: 100 mg once daily; may increase to 300 mg once daily for additional glycaemic control eGFR 30 to <60: 100 mg once daily eGFR <30: Initiation not recommended; however, patients with albuminuria >300 mg/day may continue 100 mg once daily to reduce the risk of end-stage kidney disease, doubling of serum creatinine, cardiovascular death and hospitalization for HF	eGFR ≥45: Recommended starting dose of 5 mg once daily to improve glycaemic control; 10 mg once daily for all other indications eGFR 25 to <45: 10 mg once daily eGFR <25: Initiation not recommended; may continue 10 mg once daily to reduce the risk of eGFR decline, end-stage kidney disease, cardiovascular death and hospitalization for HF	eGFR ≥30: No dose adjustment required eGFR <30: Use not recommended solely for improvement of glycaemic control Data are insufficient to provide dosing recommendations in patients: with T2D and established cardiovascular disease with eGFR <30 with HF and eGFR <20	eGFR ≥45: No dose adjustment required eGFR <45: Use not recommended

*CKD = chronic kidney disease; eGFR = estimated glomerular filtration rate; HF = heart failure; MACE = major adverse cardiovascular events; NYHA = New York Heart Association; T2D = type 2 diabetes mellitus.*

**Table 5:** Key monitoring and risk mitigation strategies for sodium–glucose co-transporter-2 inhibitors^67^

Consideration	Monitoring and/or risk mitigation strategies
Genital mycotic infections	Counsel on genital hygiene (e.g. regular bathing and wearing clean undergarments)
Hypoglycaemia	Adjust background glucose-lowering agents (e.g. insulin and/or sulfonylureas) as appropriate
Volume depletion	Proactive dose reduction of diuretics in patients at high risk for hypovolaemia Hold SGLT2 inhibitors during illness
Diabetic ketoacidosis	Educate about signs/symptoms to facilitate early recognition Monitor blood or urine ketones for very high risk Institute a sick day protocol Maintain at least low-dose insulin in insulin-requiring individuals

In sum, a patient-centred approach should guide the selection of SGLT2 inhibitors for patients with and without T2D to improve cardiovascular and kidney outcomes, irrespective of HbA1c. Key considerations in the setting of T2D include 1) the risks of hypoglycaemia, ASCVD, CKD and HF; 2) the impact on body weight; 3) their costs and access; 4) their side effects and tolerability considerations; and 5) patient preference. Patients’ medication regimen and medication-taking behaviour should be re-evaluated regularly (every 3–6 months) and adjusted as needed to incorporate patient-specific factors that may impact the choice of treatment.^8^ Furthermore, based on current guidelines, SGLT2 inhibitors should also be considered to improve HF outcomes in patients without T2D when clinically appropriate.

## Conclusions

Recommendations for using SGLT2 inhibitors in patients with and without T2D are evolving rapidly. As new data are published demonstrating the benefits of this drug class on heart and kidney outcomes, SGLT2 inhibitors are recommended not only as glucose-lowering agents, but also for their organ protective effects. They reduce risks for ASCVD, HF and CKD in the setting of T2D. Additionally, current data suggest the cardiovascular and kidney benefits of SGLT2 inhibition may extend to patients without T2D.

SGLT2 inhibitors likely work through multiple mechanisms beyond their beneficial effects on glycaemia, blood pressure and body weight. Ultimately, SGLT2 inhibitors are now considered standard of care for patients with T2D and comorbid cardiovascular and kidney disease.
